# Genetically Diverse Highly Pathogenic Avian Influenza A(H5N1/H5N8) Viruses among Wild Waterfowl and Domestic Poultry, Japan, 2021

**DOI:** 10.3201/eid2807.212586

**Published:** 2022-07

**Authors:** Kosuke Okuya, Junki Mine, Kaori Tokorozaki, Isshu Kojima, Mana Esaki, Kohtaro Miyazawa, Ryota Tsunekuni, Saki Sakuma, Asuka Kumagai, Yoshihiro Takadate, Yuto Kikutani, Tsutomu Matsui, Yuko Uchida, Makoto Ozawa

**Affiliations:** Kagoshima University, Kagoshima, Japan (K. Okuya, I. Kojima, M. Esaki, M. Ozawa);; National Agriculture and Food Research Organization, Tsukuba, Japan (J. Mine, K. Miyazawa, R. Tsunekuni, S. Sakuma, A. Kumagai, Y. Takadate, Y. Uchida);; Kagoshima Crane Conservation Committee, Izumi, Japan (K. Tokorozaki, T. Matsui, M. Ozawa);; Ministry of Agriculture, Forestry and Fisheries, Tokyo, Japan (Y. Kikutani)

**Keywords:** influenza, viruses, zoonoses, respiratory infections, high pathogenicity avian influenza virus, H5 subtype, genetic characteristics, Japan

## Abstract

Genetic analyses of highly pathogenic avian influenza H5 subtype viruses isolated from the Izumi Plain, Japan, revealed cocirculation of 2 genetic groups of clade 2.3.4.4b viruses among migratory waterfowl. Our findings demonstrate that both continuous surveillance and timely information sharing of avian influenza viruses are valuable for rapid risk assessment.

Highly pathogenic avian influenza (HPAI) viruses are known to have zoonotic potential (*1*). Therefore, global surveillance for HPAI virus in in domestic poultry and wild waterfowl is essential for assessing potential risk for both public and animal health. 

During winter 2020–21, an emerging HPAI A(H5N8) virus caused outbreaks in wild birds and domestic poultry in East Asia (*2*–*5*). Genetic and phylogenetic analyses revealed that the H5 hemagglutinin (HA) genes of H5N8 virus belonged to clade 2.3.4.4b and were divided into 2 genetic groups, G1 and G2 (*6*). The G1 viruses showed high genetic similarity with the HPAI H5N8 viruses circulating in Europe during winter 2019–20 (*7*), but the G2 viruses concurrently caused HPAI outbreaks in Europe and Asia during winter 2020–21 (*8*). We report the genetic characteristics of 4 HPAI viruses isolated from the Izumi Plain, Japan, in November 2021.

## The Study

**Figure 1:**
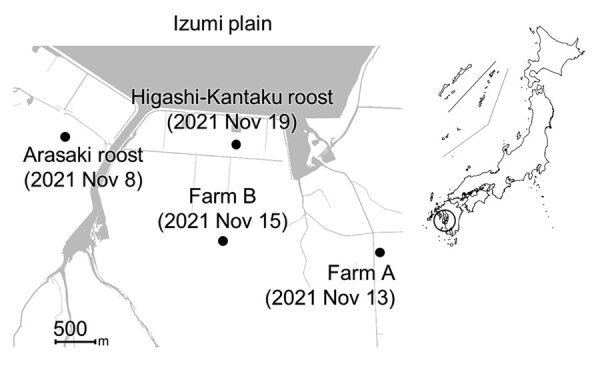
Locations on the Izumi Plain, Japan, where highly pathogenic avian influenza A(H5N1/H5N8) viruses were detected among wild waterfowl roosts and domestic poultry farms, 2021. Dots indicate location and date of avian influenza A detection. Inset map shows location of Izumi Plain in Japan.

During routine winter 2021–22 avian influenza virus (AIV) surveillance, we detected HPAI virus in environmental water samples collected from a crane roosting site in the Arasaki area of the Izumi Plain on November 8, 2021 (Figure 1). We inoculated embryonated chicken eggs (*9*) with those water samples and isolated AIVs of mixed subtypes, most likely due to co-inoculation with multiple AIVs. We could not determine the neuraminidase (NA) subtype due to the mixed virus populations. However, using the MinION Mk1B nanopore sequencer (Oxford Nanopore Technologies, https://nanoporetech.com), as described previously (*10*), we confirmed that 1 virus isolate, A/environment/Kagoshima/KU-ngrB4/2021 (mixed), was an HPAI H5 subtype (Table). In addition to the H5 HA gene, we detected HA genes of H3 and H4 subtypes and NA genes of N6 and N8 subtypes from A/environment/Kagoshima/KU-ngrB4/2021 (mixed). Based on our BLAST analysis (https://blast.ncbi.nlm.nih.gov), HA gene segments of the detected H3 virus showed the highest similarity to those from H3N8 virus A/duck/Mongolia/MN18-14/2018 (97.65%) and the H4 virus showed the highest similarity to HA genes from H4N2 virus A/duck/Bangladesh/41653/2019 (98.85%). The NA gene segment of H3N6 virus showed the highest similarity to those from H3N6 virus A/duck/Mongolia/MN18-1/2018 (98.8%) and the NA gene segment of H3N8 virus showed highest similarity to H5N8 virus A/water/Tottori/NK1201-2/2021 (99.36%). In contrast to the HA and NA gene segments, we detected only single nucleotide sequences in the remaining 6 gene segments. The closest relatives of these 6 gene segments all were derived from recent HPAI H5N8 viruses. In addition, the H5 HA gene, N8 NA gene, and remaining gene segments from 2 virus isolates, A/environment/Kagoshima/KU-ngrB4/2021 (mixed) and A/hooded crane/Kagoshima/KU-5T/2021 (H5N8), were nearly identical (>99.8%).

**Table:** Collection date, GISAID accession numbers, closest genetic relatives, and percentage genetic identity compared with other viruses among highly pathogenic avian influenza A(H5N1/H5N8) viruses detected in the Izumi Plain, Japan, 2021*

Isolated virus	Collection date	Gene	Accession no.	Closest relative†	% Identity
A/environment/Kagoshima/ KU-ngrB4/2021 (mixed)	2021 Nov 8	HA	EPI1933367	A/environment sample/China/TZ001/2021 (H5N8)	99.47
A/chicken/Kagoshima/ 21A6T/2021 (H5N1)	2021 Nov 13	PB2	EPI1940236	A/duck/Bangladesh/37630/2019 (H10N4)	97.72
		PB1	EPI1940237	A/layer hen/Slovakia/A-chicken-Slovakia-Pah_14–2020 /2020 (H5N8)	98.20
		PA	EPI1940238	A/chicken/Nigeria/VRD21–98_21VIR2288–6/2021 (H5N1)	98.05
		HA	EPI1933663	A/chicken/Nigeria/VRD21–43_21VIR2288–4/2021 (H5N8)	98.71
		NP	EPI1940239	A/teal/Egypt/MB-D-487OP/2016 (H7N3)	98.20
		NA	EPI1940240	A/chicken/Nigeria/VRD21–109_21VIR2370–425/2021 (H5N1)	97.83
		M	EPI1940241	A/*Cygnus columbianus*/Hubei/56/2020 (H5N8)	99.29
		NSP	EPI1940242	A/environment/Bangladesh/42635/2020 (H10N7)	99.28
A/chicken/Kagoshima/ B3T/2021 (H5N8)	2021 Nov 15	PB2	EPI1933684	A/environment sample/China/TZ001/2021 (H5N8)	99.61
		PB1	EPI1933685	A/environment sample/China/TZ001/2021 (H5N8)	99.30
		PA	EPI1933683	A/*Cygnus columbianus*/Hubei/116/2020 (H5N8)	99.40
		HA	EPI1933687	A/environment sample/China/TZ001/2021 (H5N8)	99.53
		NP	EPI1933680	A/environment sample/China/TZ001/2021 (H5N8)	99.67
		NA	EPI1933686	A/*Cygnus columbianus*/Hubei/51/2020 (H5N8)	99.15
		M	EPI1933682	A/*Cygnus columbianus*/Hubei/56/2020 (H5N8)	99.59
		NSP	EPI1933681	A/environment sample/China/TZ001/2021 (H5N8)	99.64
A/hooded crane/Kagoshima/ KU-5T/2021 (H5N8)	2021 Nov 19	PB2	EPI1933368	A/environment sample/China/TZ001/2021 (H5N8)	99.56
		PB1	EPI1933369	A/environment sample/China/TZ001/2021 (H5N8)	99.30
		PA	EPI1933370	A/*Cygnus columbianus*/Hubei/116/2020 (H5N8)	99.49
		HA	EPI1933371	A/environment sample/China/TZ001/2021 (H5N8)	99.47
		NP	EPI1933372	A/environment sample/China/TZ001/2021 (H5N8)	99.67
		NA	EPI1933373	A/*Cygnus columbianus*/Hubei/51/2020 (H5N8)	99.09
		M	EPI1933374	A/*Cygnus columbianus*/Hubei/56/2020 (H5N8)	99.39
		NSP	EPI1933375	A/environment sample/China/TZ001/2021 (H5N8)	99.76

*GISAID, https://www.gisaid.org. HA, hemagglutinin; M, matrix; NA, neuraminidase; NP, nucleoprotein; NSP, nonstructural protein; PA, polymerase; PB1, polymerase basic 1; PB2, polymerase basic 2. †Representative viruses with the highest nucleotide identity retrieved from the NCBI GenBank (https://www.ncbi.nlm.nih.gov/genbank) database on December 3, 2021.

A layer chicken farm, farm A, reported unusual mortality among chickens on November 13, 2021 (Figure 1). We used the Miseq platform (Illumina, https://www.illumina.com) to perform viral genome sequencing on isolates from farm A and found an H5N1 virus isolate, A/chicken/Kagoshima/21A6T/2021, possessed the high pathogenicity H5 HA gene (Table). After HPAI virus outbreak notification from farm A, local authorities conducted legally mandated urgent investigations at 25 chicken farms located within 3 km of the farm. Subsequent investigations discovered another HPAI virus outbreak at a layer chicken farm, farm B (Figure 1), before increased poultry mortality occurred there on November 15, 2021. Of note, Miseq viral genome sequencing revealed that the farm B virus was an HPAI H5N8 virus, A/chicken/Kagoshima/B3T/2021 (Table).

On November 19, 2021, a hooded crane (*Grus monacha*) was found dead at a second roosting area in Higashi-Kantaku (Figure 1). Using a tracheal swab sample from the dead crane, we isolated another HPAI H5N8 virus, A/hooded crane/Kagoshima/KU-5T/2021, and sequenced its genome by using the MinION Mk1B (Table). Thus, we detected 4 HPAI H5 viruses from different sources within a 5-km radius in only 12 days.

Using BLAST, we analyzed the nucleotide sequences of all 8 gene segments from each virus isolate (Table). The sequences sharing the highest nucleotide identity with the polymerase basic 2, nucleoprotein, and nonstructural protein gene segments from 1 isolate, A/chicken/Kagoshima/21A6T/2021 (H5N1), were sequences from low pathogenicity avian influenza (LPAI) viruses isolated from wild ducks (Table). These findings indicate that the HPAI H5N1 virus we detected is a genetic reassortant recently generated between HPAI and LPAI viruses.

In contrast, each gene segment from isolates A/chicken/Kagoshima/B3T/2021 (H5N8) and A/hooded crane/Kagoshima/KU-5T/2021 (H5N8) shared relatively high similarity (>99%) with those from HPAI H5N8 viruses isolated from a tundra swan (*Cygnus columbianus*) or environmental samples collected in China during the 2019–20 and 2020–21 winter seasons (Table). In addition, nucleotide sequences of all 8 gene segments from both isolates were almost identical to each other (Appendix Figure 1). These results suggest that the HPAI outbreak on farm B was caused by HPAI H5N8 virus progenies that have been detected in migratory waterfowl in East Asia since 2019. Of note, the HPAI viruses we detected did not demonstrate any amino acid substitutions related to mammalian adaptation, such as a single amino acid substitution of glutamine to lysine at position 591 (Q591K), E627K, or D701N in the polymerase basic 2 protein (*11*–*13*); nor Q226L, N224K, or G228S in the H5 HA protein (*14*,*15*).

**Figure 2:**
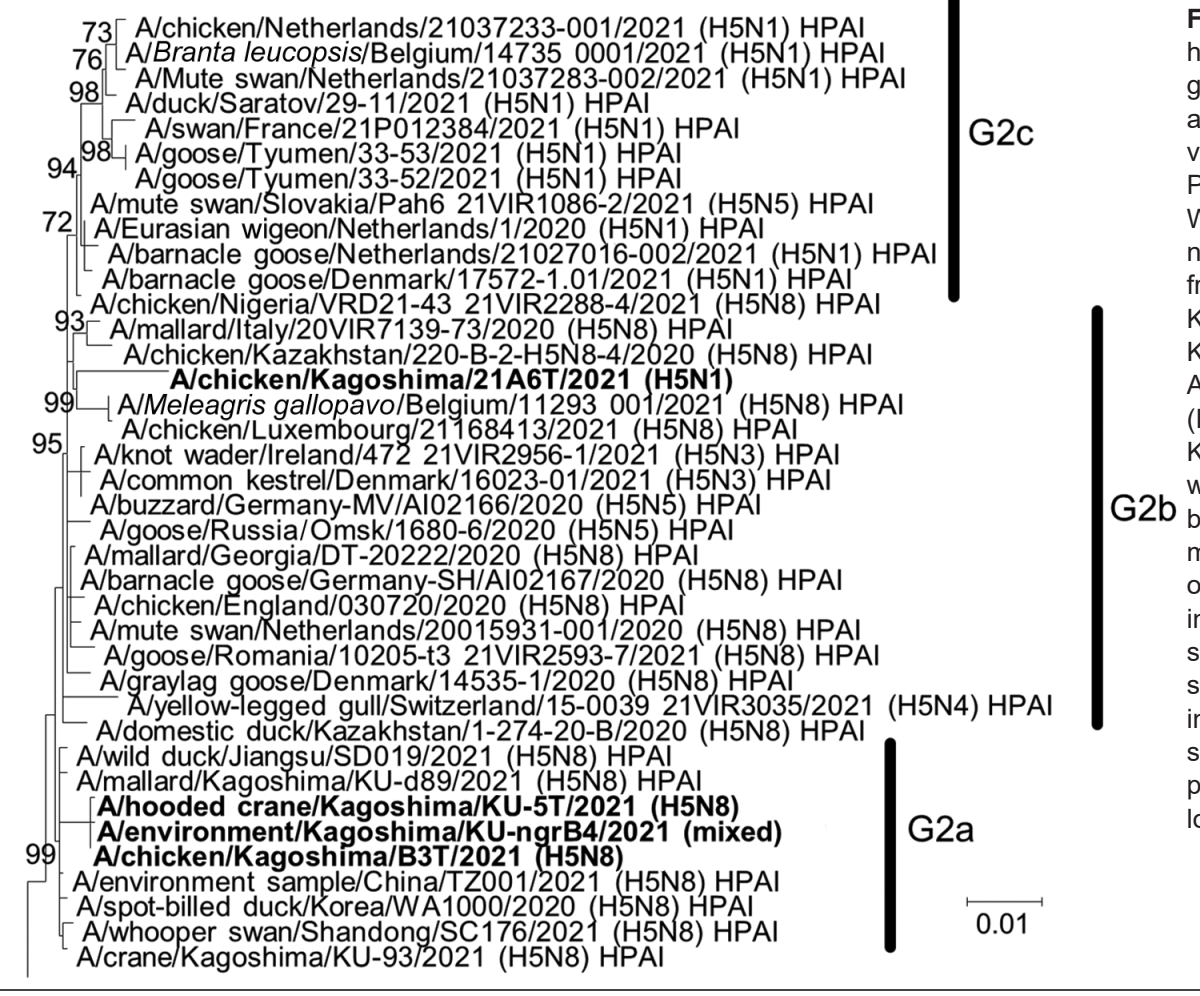
Phylogenetic tree of hemagglutinin genes of genetic group 2 (G2) of highly pathogenic avian influenza A(H5N1/H5N8) viruses isolated on the Izumi Plain, Japan, in November 2021. We phylogenetically analyzed the nucleotide sequences of the genes from A/environment/Kagoshima/KU-ngrB4/2021 (mixed), A/chicken/Kagoshima/21A6T/2021 (H5N1), A/chicken/Kagoshima/B3T/2021 (H5N8), and A/hooded crane/Kagoshima/KU-5T/2021 (H5N8) with representative counterparts by using the maximum-likelihood method with a bootstrapping set of 1,000 replicates. Bold text indicates viruses isolated in this study. Bootstrap values >70% are shown at the nodes. Scale bar indicates the number of nucleotide substitutions per site. HPAI, highly pathogenic avian influenza; LPAI, low pathogenicity avian influenza.

The phylogenetic tree of the H5 HA gene revealed that all 4 HPAI viruses we detected belong to genetic group G2 of clade 2.3.4.4b (Figure 2; Appendix Figure 2). The H5 HA gene from A/chicken/Kagoshima/21A6T/2021 (H5N1) comprises a cluster with HA genes from HPAI H5N8 viruses detected during the 2021–22 winter season in Europe, and we tentatively designated this cluster as subgroup G2b (Figure 2). These results suggest that genetically similar HPAI H5 viruses simultaneously invaded Europe and East Asia during the 2021–22 winter season, possibly because the migratory waterfowl populations flying to each region shared the same breeding areas during summer 2021. The H5N1 NA gene tree indicated that the closest ancestors might be LPAI viruses (Appendix Figure 1, panel A), but genetic similarity to recent HPAI viruses detected in Africa and Europe was also evident (Table). Phylogenetic trees of the remaining 6 H5N1 genes also attested that A/chicken/Kagoshima/21A6T/2021 (H5N1) is a likely genetic reassortant recently generated between HPAI and LPAI viruses (Appendix Figure 1, panels B–G).

Unlike A/chicken/Kagoshima/21A6T/2021 (H5N1), the gene constellations of A/chicken/Kagoshima/B3T/2021 (H5N8) and A/hooded crane/Kagoshima/KU-5T/2021 (H5N8) were the same as HPAI H5N8 viruses detected during the 2020–21 winter season in East Asia, as we noted in subgroup G2a (Figure 2; Appendix Figure 1, panels B–H). These results suggest that A/chicken/Kagoshima/21A6T/2021 (H5N1) and the 3 other HPAI viruses we detected evolved individually among migratory waterfowl.

## Conclusions

The results of this study, together with the contemporary HPAI outbreaks in other regions, including neighboring countries and in Europe, suggest that the HPAI H5N8 viruses isolated at farm B were introduced from migratory waterfowl overwintering on the same plain. Genetic analyses also revealed that 2 genetic subgroups of HPAI H5N1/H5N8 viruses, G2a and G2b, cocirculated among the migratory waterfowl on the Izumi Plain. The HA genes from the HPAI H5 viruses isolated in Europe during the 2021–22 winter season formed a single cluster that was distinct from G2a and G2b; because HPAI viruses belonging to this cluster have not yet been isolated in Asia, we tentatively designated this genetic subgroup as G2c (Figure 2). This subgroup, which has been causing HPAI outbreaks in Europe since October 2021, could be introduced into East Asia. 

In conclusion, we isolated and analyzed 4 HPAI H5N1/H5N8 viruses of clade 2.3.4.4b from the Izumi Plain, Japan, and found potential reassortment between HPAI and LPAI viruses. Our findings support the need for continuous surveillance and timely information sharing for rapid assessment of the potential risks to public and animal health.

Appendix Additional information on highly pathogenic avian influenza A(H5N1/H5N8) viruses detected among wild waterfowl and domestic poultry, Japan, 2021.
