# Supplementary material for: Genetically Diverse Highly Pathogenic Avian Influenza A(H5N1/H5N8) Viruses among Wild Waterfowl and Domestic Poultry, Japan, 2021
Source: Emerg Infect Dis. 2022 Jul;28(7):1451–5. doi: 10.3201/eid2807.212586 (PMC9239871; doi:10.3201/eid2807.212586)
Supplement: Appendix — Additional information on highly pathogenic avian influenza A(H5N1/H5N8) viruses detected among wild waterfowl and domestic poultry, Japan, 2021. [file 21-2586-Techapp-s1.pdf]

Article DOI: https://doi.org/10.3201/eid2807.212586

Genetically Diverse Highly Pathogenic Avian Influenza A(H5N1/H5N8) Viruses among Wild Waterfowl and Domestic Poultry, Japan, 2021

Appendix

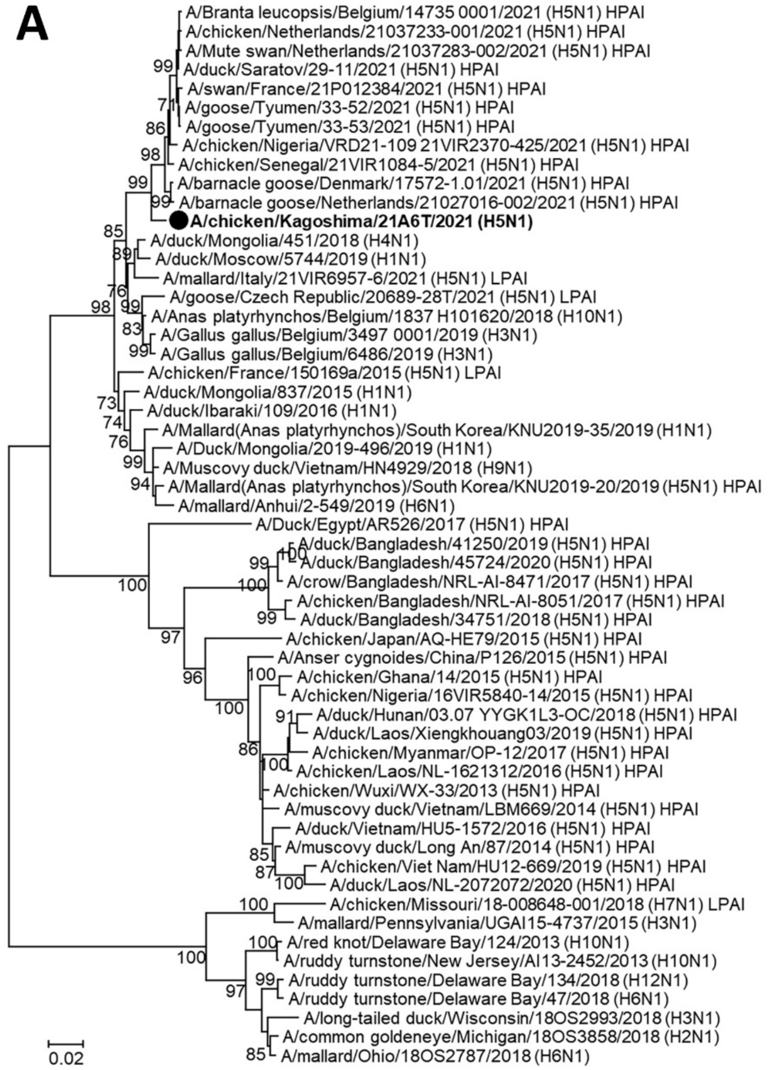

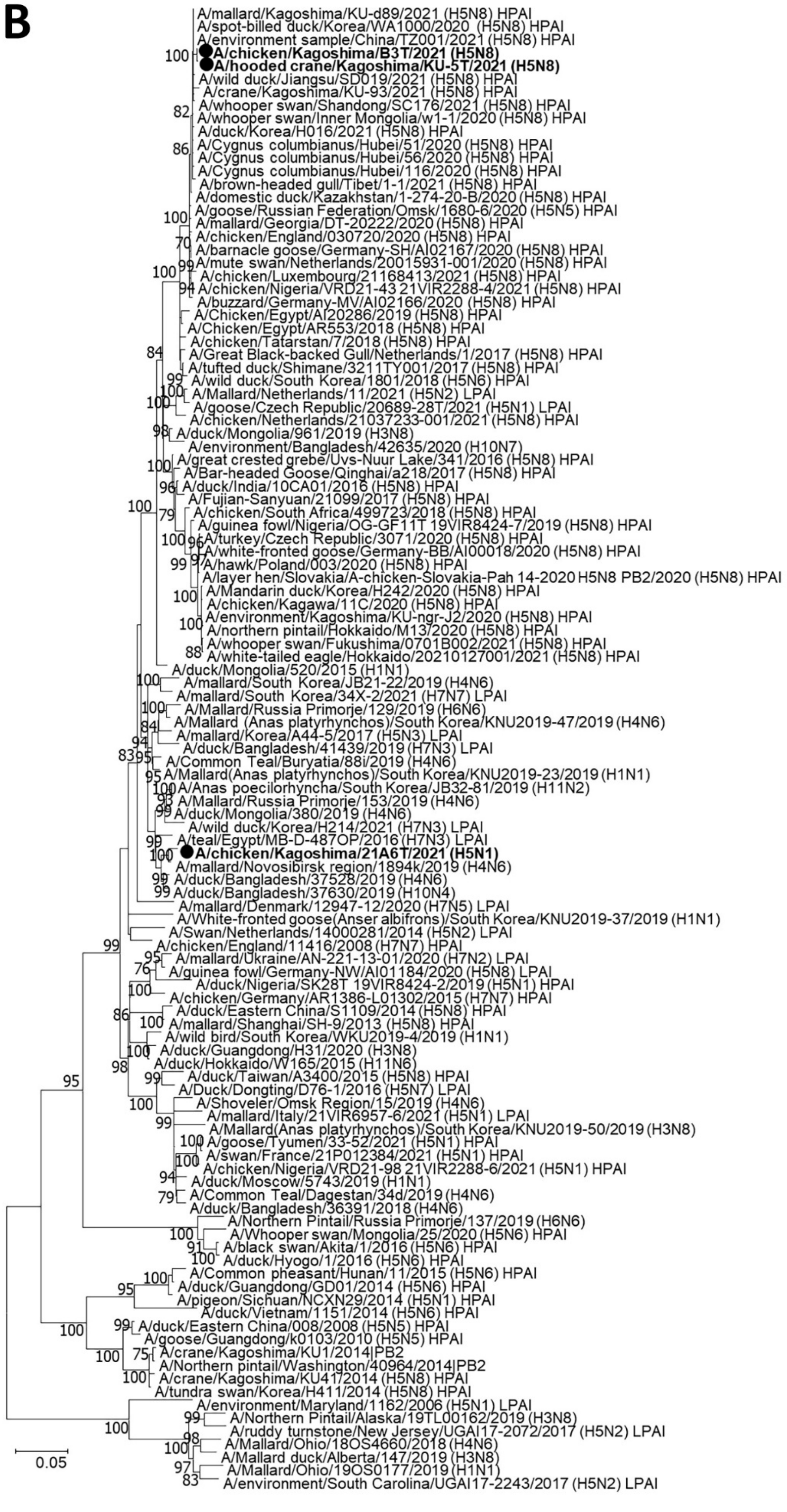
B
A/mallard/Kagoshima/KU-d89/2021 (H5N8) HPAI
A/spot-billed duck/Korea/WA1000/2020 (H5N8) HPAI
A/environment sample/China/TZ001/2021 (H5N8) HPAI
A/chicken/Kagoshima/B3T/2021 (H5N8)
A/hooded crane/Kagoshima/KU-5T/2021 (H5N8)
A/wild duck/Jiangsu/SD019/2021 (H5N8) HPAI
A/crane/Kagoshima/KU-93/2021 (H5N8) HPAI
A/whooper swan/Shandong/SC176/2021 (H5N8) HPAI
A/whooper swan/Inner Mongolia/w1-1/2020 (H5N8) HPAI
A/duck/Korea/H016/2021 (H5N8) HPAI
A/Cygnus columbianus/Hubei/51/2020 (H5N8) HPAI
A/Cygnus columbianus/Hubei/56/2020 (H5N8) HPAI
A/Cygnus columbianus/Hubei/116/2020 (H5N8) HPAI
A/brown-headed gull/Tibet/1-1/2021 (H5N8) HPAI
A/domestic duck/Kazakhstan/1-274-20-B/2020 (H5N8) HPAI
A/goose/Russian Federation/Omsk/1680-6/2020 (H5N5) HPAI
A/mallard/Georgia/DT-20222/2020 (H5N8) HPAI
A/chicken/England/030720/2020 (H5N8) HPAI
A/barnacle goose/Germany-SH/AI02167/2020 (H5N8) HPAI
A/mute swan/Netherlands/20015931-001/2020 (H5N8) HPAI
A/chicken/Luxembourg/21168413/2021 (H5N8) HPAI
A/chicken/Nigeria/VRD21-43 21VIR2288-4/2021 (H5N8) HPAI
A/buzzard/Germany-MV/AI02166/2020 (H5N8) HPAI
A/Chicken/Egypt/AI20286/2019 (H5N8) HPAI
A/Chicken/Egypt/AR553/2018 (H5N8) HPAI
A/chicken/Tatarstan/7/2018 (H5N8) HPAI
A/Great Black-backed Gull/Netherlands/1/2017 (H5N8) HPAI
A/tufted duck/Shimane/3211TY001/2017 (H5N8) HPAI
A/wild duck/South Korea/1801/2018 (H5N6) HPAI
A/Mallard/Netherlands/11/2021 (H5N2) LPAI
A/goose/Czech Republic/20689-28T/2021 (H5N1) LPAI
A/chicken/Netherlands/21037233-001/2021 (H5N8) HPAI
A/duck/Mongolia/961/2019 (H3N8)
A/environment/Bangladesh/42635/2020 (H10N7)
A/great crested grebe/Uvs-Nuur Lake/341/2016 (H5N8) HPAI
A/Bar-headed Goose/Qinghai/a218/2017 (H5N8) HPAI
A/duck/India/10CA01/2016 (H5N8) HPAI
A/Fujian-Sanyuan/21099/2017 (H5N8) HPAI
A/chicken/South Africa/499723/2018 (H5N8) HPAI
A/guinea fowl/Nigeria/OG-GF11T 19VIR8424-7/2019 (H5N8) HPAI
A/turkey/Czech Republic/3071/2020 (H5N8) HPAI
A/white-fronted goose/Germany-BB/AI00018/2020 (H5N8) HPAI
A/hawk/Poland/003/2020 (H5N8) HPAI
A/layer hen/Slovakia/A-chicken-Slovakia-Pah 14-2020 H5N8 PB2/2020 (H5N8) HPAI
A/Mandarin duck/Korea/H242/2020 (H5N8) HPAI
A/chicken/Kagawa/11C/2020 (H5N8) HPAI
A/environment/Kagoshima/KU-ngr-J2/2020 (H5N8) HPAI
A/northern pintail/Hokkaido/M13/2020 (H5N8) HPAI
A/whooper swan/Fukushima/0701B002/2021 (H5N8) HPAI
A/white-tailed eagle/Hokkaido/20210127001/2021 (H5N8) HPAI
A/duck/Mongolia/520/2015 (H1N1)
A/mallard/South Korea/JB21-22/2019 (H4N6)
A/mallard/South Korea/34X-2/2021 (H7N7) LPAI
A/Mallard/Russia Primorje/129/2019 (H6N6)
A/Mallard (Anas platyrhynchos)/South Korea/KNU2019-47/2019 (H4N6)
A/mallard/Korea/A44-5/2017 (H5N3) LPAI
A/duck/Bangladesh/41439/2019 (H7N3) LPAI
A/Common Teal/Buryatia/88i/2019 (H4N6)
A/Mallard(Anas platyrhynchos)/South Korea/KNU2019-23/2019 (H1N1)
A/Anas poecilorhyncha/South Korea/JB32-81/2019 (H11N2)
A/Mallard/Russia Primorje/153/2019 (H4N6)
A/duck/Mongolia/380/2019 (H4N6)
A/wild duck/Korea/H214/2021 (H7N3) LPAI
A/teal/Egypt/MB-D-487OP/2016 (H7N3) LPAI
A/chicken/Kagoshima/21A6T/2021 (H5N1)
A/mallard/Novosibirsk region/1894k/2019 (H4N6)
A/duck/Bangladesh/37528/2019 (H4N6)
A/duck/Bangladesh/37630/2019 (H10N4)
A/mallard/Denmark/12947-12/2020 (H7N5) LPAI
A/White-fronted goose(Anser albifrons)/South Korea/KNU2019-37/2019 (H1N1)
A/Swan/Netherlands/14000281/2014 (H5N2) LPAI
A/chicken/England/11416/2008 (H7N7) HPAI
A/mallard/Ukraine/AN-221-13-01/2020 (H7N2) LPAI
A/guinea fowl/Germany-NW/AI01184/2020 (H5N8) LPAI
A/duck/Nigeria/SK28T 19VIR8424-2/2019 (H5N1) HPAI
A/chicken/Germany/AR1386-L01302/2015 (H7N7) HPAI
A/duck/Eastern China/S1109/2014 (H5N8) HPAI
A/mallard/Shanghai/SH-9/2013 (H5N8) HPAI
A/wild bird/South Korea/WKU2019-4/2019 (H1N1)
A/duck/Guangdong/H31/2020 (H3N8)
A/duck/Hokkaido/W165/2015 (H11N6)
A/duck/Taiwan/A3400/2015 (H5N8) HPAI
A/Duck/Dongting/D76-1/2016 (H5N7) LPAI
A/Shoveler/Omsk Region/15/2019 (H4N6)
A/mallard/Italy/21VIR6957-6/2021 (H5N1) LPAI
A/Mallard(Anas platyrhynchos)/South Korea/KNU2019-50/2019 (H3N8)
A/goose/Tyumen/33-52/2021 (H5N1) HPAI
A/swan/France/21P012384/2021 (H5N1) HPAI
A/chicken/Nigeria/VRD21-98 21VIR2288-6/2021 (H5N1) HPAI
A/duck/Moscow/5743/2019 (H1N1)
A/Common Teal/Dagestan/34d/2019 (H4N6)
A/duck/Bangladesh/36391/2018 (H4N6)
A/Northern Pintail/Russia Primorje/137/2019 (H6N6)
A/Whooper swan/Mongolia/25/2020 (H5N6) HPAI
A/black swan/Akita/1/2016 (H5N6) HPAI
A/duck/Hyogo/1/2016 (H5N6) HPAI
A/Common pheasant/Hunan/11/2015 (H5N6) HPAI
A/duck/Guangdong/GD01/2014 (H5N6) HPAI
A/pigeon/Sichuan/NCXN29/2014 (H5N1) HPAI
A/duck/Vietnam/1151/2014 (H5N6) HPAI
A/duck/Eastern China/008/2008 (H5N5) HPAI
A/goose/Guangdong/k0103/2010 (H5N5) HPAI
A/crane/Kagoshima/KU1/2014|PB2
A/Northern pintail/Washington/40964/2014|PB2
A/crane/Kagoshima/KU41/2014 (H5N8) HPAI
A/tundra swan/Korea/H411/2014 (H5N8) HPAI
A/environment/Maryland/1162/2006 (H5N1) LPAI
A/Northern Pintail/Alaska/19TL00162/2019 (H3N8)
A/ruddy turnstone/New Jersey/UGAI17-2072/2017 (H5N2) LPAI
A/Mallard/Ohio/18OS4660/2018 (H4N6)
A/Mallard duck/Alberta/147/2019 (H3N8)
A/Mallard/Ohio/19OS0177/2019 (H1N1)
A/environment/South Carolina/UGAI17-2243/2017 (H5N2) LPAI
0.05

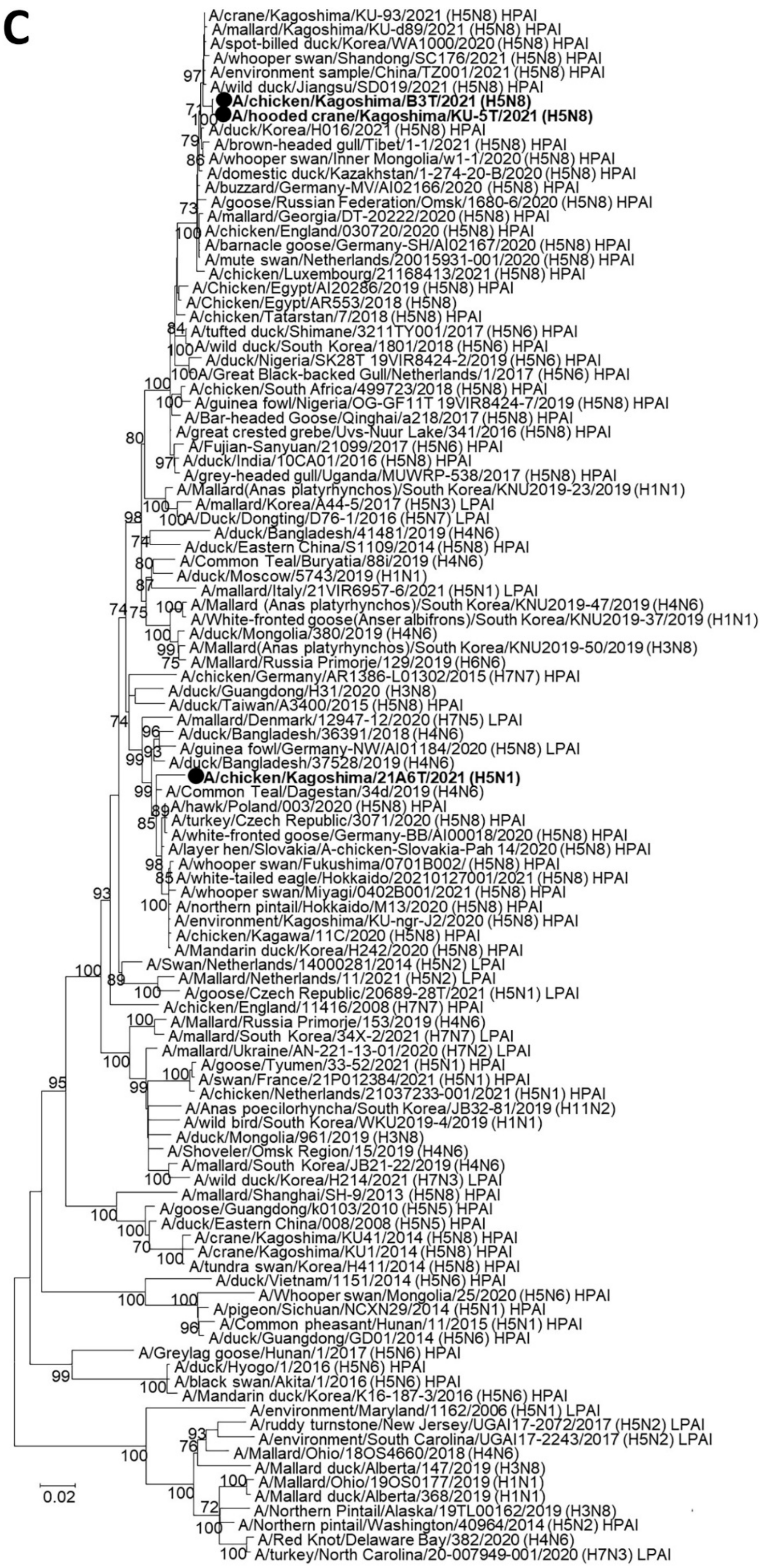
C
A/crane/Kagoshima/KU-93/2021 (H5N8) HPAI
A/mallard/Kagoshima/KU-d89/2021 (H5N8) HPAI
A/spot-billed duck/Korea/WA1000/2020 (H5N8) HPAI
A/whooper swan/Shandong/SC176/2021 (H5N8) HPAI
97
A/environment sample/China/TZ001/2021 (H5N8) HPAI
A/wild duck/Jiangsu/SD019/2021 (H5N8) HPAI
A/chicken/Kagoshima/B3T/2021 (H5N8)
71
100
A/hooded crane/Kagoshima/KU-5T/2021 (H5N8)
A/duck/Korea/H016/2021 (H5N8) HPAI
79
A/brown-headed gull/Tibet/1-1/2021 (H5N8) HPAI
86
A/whooper swan/Inner Mongolia/w1-1/2020 (H5N8) HPAI
A/domestic duck/Kazakhstan/1-274-20-B/2020 (H5N8) HPAI
A/buzzard/Germany-MV/AI02166/2020 (H5N8) HPAI
A/goose/Russian Federation/Omsk/1680-6/2020 (H5N8) HPAI
73
A/mallard/Georgia/DT-20222/2020 (H5N8) HPAI
100
A/chicken/England/030720/2020 (H5N8) HPAI
A/barnacle goose/Germany-SH/AI02167/2020 (H5N8) HPAI
A/mute swan/Netherlands/20015931-001/2020 (H5N8) HPAI
A/chicken/Luxembourg/21168413/2021 (H5N8) HPAI
A/Chicken/Egypt/AI20286/2019 (H5N8) HPAI
A/Chicken/Egypt/AR553/2018 (H5N8)
A/chicken/Tatarstan/7/2018 (H5N8) HPAI
84
A/tufted duck/Shimane/3211TY001/2017 (H5N6) HPAI
A/wild duck/South Korea/1801/2018 (H5N6) HPAI
100
A/duck/Nigeria/SK28T 19VIR8424-2/2019 (H5N6) HPAI
100
A/Great Black-backed Gull/Netherlands/1/2017 (H5N6) HPAI
100
A/chicken/South Africa/499723/2018 (H5N8) HPAI
100
A/guinea fowl/Nigeria/OG-GF11T 19VIR8424-7/2019 (H5N8) HPAI
A/Bar-headed Goose/Qinghai/a218/2017 (H5N8) HPAI
A/great crested grebe/Uvs-Nuur Lake/341/2016 (H5N8) HPAI
80
A/Fujian-Sanyuan/21099/2017 (H5N6) HPAI
97
A/duck/India/10CA01/2016 (H5N8) HPAI
A/grey-headed gull/Uganda/MUWRP-538/2017 (H5N8) HPAI
A/Mallard(Anas platyrhynchos)/South Korea/KNU2019-23/2019 (H1N1)
100
A/mallard/Korea/A44-5/2017 (H5N3) LPAI
98
100
A/Duck/Dongting/D76-1/2016 (H5N7) LPAI
A/duck/Bangladesh/41481/2019 (H4N6)
74
A/duck/Eastern China/S1109/2014 (H5N8) HPAI
80
A/Common Teal/Buryatia/88i/2019 (H4N6)
A/duck/Moscow/5743/2019 (H1N1)
87
A/mallard/Italy/21VIR6957-6/2021 (H5N1) LPAI
74
75
100
A/Mallard (Anas platyrhynchos)/South Korea/KNU2019-47/2019 (H4N6)
A/White-fronted goose(Anser albifrons)/South Korea/KNU2019-37/2019 (H1N1)
100
A/duck/Mongolia/380/2019 (H4N6)
99
A/Mallard(Anas platyrhynchos)/South Korea/KNU2019-50/2019 (H3N8)
75
A/Mallard/Russia Primorje/129/2019 (H6N6)
A/chicken/Germany/AR1386-L01302/2015 (H7N7) HPAI
A/duck/Guangdong/H31/2020 (H3N8)
A/duck/Taiwan/A3400/2015 (H5N8) HPAI
74
A/mallard/Denmark/12947-12/2020 (H7N5) LPAI
96
A/duck/Bangladesh/36391/2018 (H4N6)
93
A/guinea fowl/Germany-NW/AI01184/2020 (H5N8) LPAI
99
A/duck/Bangladesh/37528/2019 (H4N6)
A/chicken/Kagoshima/21A6T/2021 (H5N1)
99
A/Common Teal/Dagestan/34d/2019 (H4N6)
A/hawk/Poland/003/2020 (H5N8) HPAI
89
A/turkey/Czech Republic/3071/2020 (H5N8) HPAI
85
A/white-fronted goose/Germany-BB/AI00018/2020 (H5N8) HPAI
A/layer hen/Slovakia/A-chicken-Slovakia-Pah 14/2020 (H5N8) HPAI
98
A/whooper swan/Fukushima/0701B002/ (H5N8) HPAI
85
A/white-tailed eagle/Hokkaido/20210127001/2021 (H5N8) HPAI
93
A/whooper swan/Miyagi/0402B001/2021 (H5N8) HPAI
100
A/northern pintail/Hokkaido/M13/2020 (H5N8) HPAI
A/environment/Kagoshima/KU-ngr-J2/2020 (H5N8) HPAI
A/chicken/Kagawa/11C/2020 (H5N8) HPAI
A/Mandarin duck/Korea/H242/2020 (H5N8) HPAI
A/Swan/Netherlands/14000281/2014 (H5N2) LPAI
100
89
A/Mallard/Netherlands/11/2021 (H5N2) LPAI
100
A/goose/Czech Republic/20689-28T/2021 (H5N1) LPAI
A/chicken/England/11416/2008 (H7N7) HPAI
100
A/Mallard/Russia Primorje/153/2019 (H4N6)
A/mallard/South Korea/34X-2/2021 (H7N7) LPAI
A/mallard/Ukraine/AN-221-13-01/2020 (H7N2) LPAI
100
A/goose/Tyumen/33-52/2021 (H5N1) HPAI
100
A/swan/France/21P012384/2021 (H5N1) HPAI
95
99
A/chicken/Netherlands/21037233-001/2021 (H5N1) HPAI
A/Anas poecilorhyncha/South Korea/JB32-81/2019 (H11N2)
A/wild bird/South Korea/WKU2019-4/2019 (H1N1)
A/duck/Mongolia/961/2019 (H3N8)
A/Shoveler/Omsk Region/15/2019 (H4N6)
A/mallard/South Korea/JB21-22/2019 (H4N6)
100
A/wild duck/Korea/H214/2021 (H7N3) LPAI
A/mallard/Shanghai/SH-9/2013 (H5N8) HPAI
A/goose/Guangdong/k0103/2010 (H5N5) HPAI
100
100
A/duck/Eastern China/008/2008 (H5N5) HPAI
A/crane/Kagoshima/KU41/2014 (H5N8) HPAI
70
100
A/crane/Kagoshima/KU1/2014 (H5N8) HPAI
A/tundra swan/Korea/H411/2014 (H5N8) HPAI
A/duck/Vietnam/1151/2014 (H5N6) HPAI
100
100
A/Whooper swan/Mongolia/25/2020 (H5N6) HPAI
A/pigeon/Sichuan/NCXN29/2014 (H5N1) HPAI
96
A/Common pheasant/Hunan/11/2015 (H5N1) HPAI
A/duck/Guangdong/GD01/2014 (H5N6) HPAI
A/Greylag goose/Hunan/1/2017 (H5N6) HPAI
A/duck/Hyogo/1/2016 (H5N6) HPAI
99
A/black swan/Akita/1/2016 (H5N6) HPAI
100
A/Mandarin duck/Korea/K16-187-3/2016 (H5N6) HPAI
A/environment/Maryland/1162/2006 (H5N1) LPAI
93
A/ruddy turnstone/New Jersey/UGAI17-2072/2017 (H5N2) LPAI
A/environment/South Carolina/UGAI17-2243/2017 (H5N2) LPAI
76
A/Mallard/Ohio/18OS4660/2018 (H4N6)
100
A/Mallard duck/Alberta/147/2019 (H3N8)
100
A/Mallard/Ohio/19OS0177/2019 (H1N1)
0.02
100
A/Mallard duck/Alberta/368/2019 (H1N1)
72
A/Northern Pintail/Alaska/19TL00162/2019 (H3N8)
100
A/Northern pintail/Washington/40964/2014 (H5N2) HPAI
A/Red Knot/Delaware Bay/382/2020 (H4N6)
100
A/turkey/North Carolina/20-007949-001/2020 (H7N3) LPAI

D

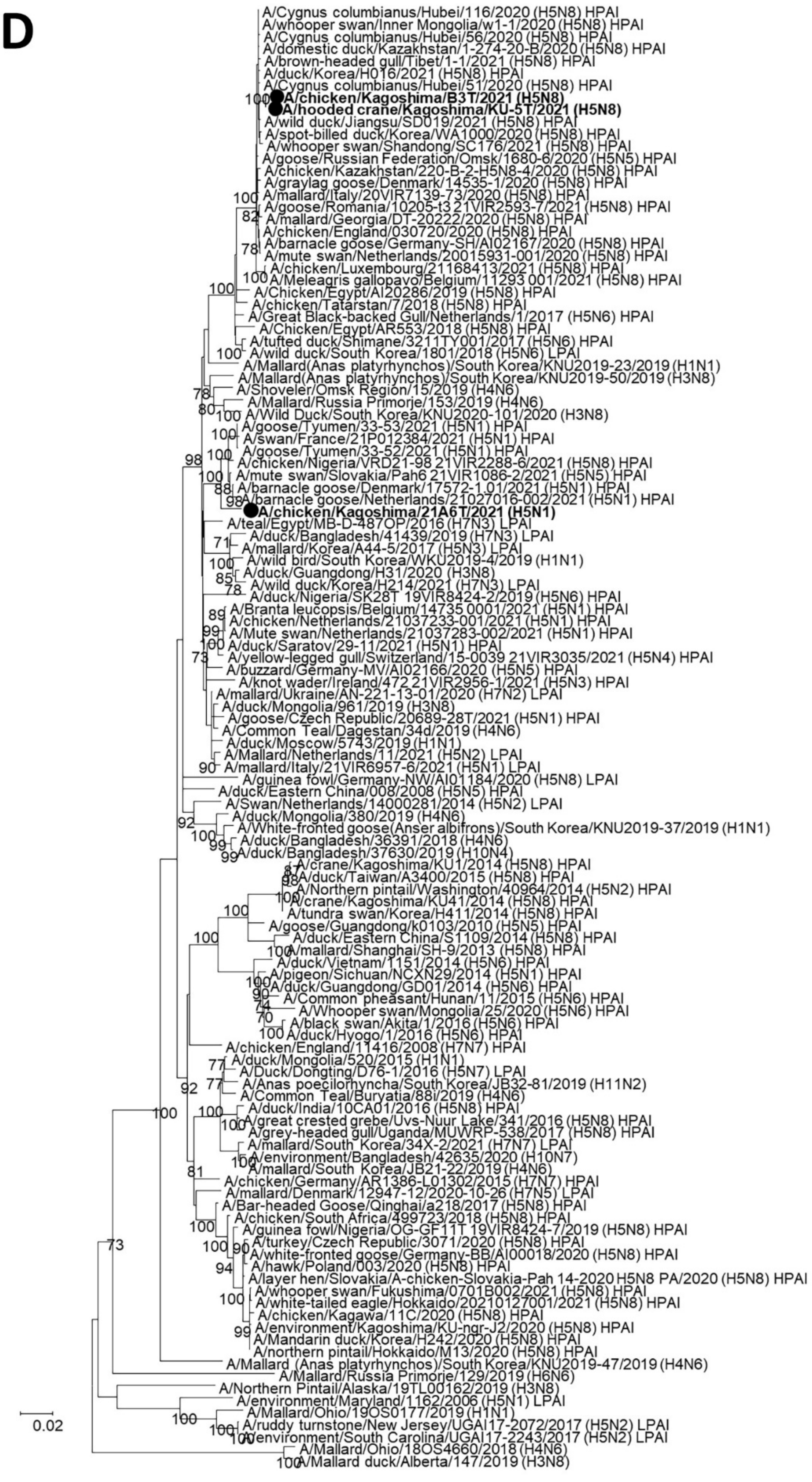
A/Cygnus columbianus/Hubei/116/2020 (H5N8) HPAI
A/whooper swan/Inner Mongolia/w1-1/2020 (H5N8) HPAI
A/Cygnus columbianus/Hubei/56/2020 (H5N8) HPAI
A/domestic duck/Kazakhstan/1-274-20-B/2020 (H5N8) HPAI
A/brown-headed gull/Tibet/1-1/2021 (H5N8) HPAI
A/duck/Korea/H016/2021 (H5N8) HPAI
A/Cygnus columbianus/Hubei/51/2020 (H5N8) HPAI
A/chicken/Kagoshima/B3T/2021 (H5N8)
A/hooded crane/Kagoshima/KU-5T/2021 (H5N8)
A/wild duck/Jiangsu/SD019/2021 (H5N8) HPAI
A/spot-billed duck/Korea/WA1000/2020 (H5N8) HPAI
A/whooper swan/Shandong/SC176/2021 (H5N8) HPAI
A/goose/Russian Federation/Omsk/1680-6/2020 (H5N5) HPAI
A/chicken/Kazakhstan/220-B-2-H5N8-4/2020 (H5N8) HPAI
A/graylag goose/Denmark/14535-1/2020 (H5N8) HPAI
A/mallard/Italy/20VIR7139-73/2020 (H5N8) HPAI
A/goose/Romania/10205-t3 21VIR2593-7/2021 (H5N8) HPAI
A/mallard/Georgia/DT-20222/2020 (H5N8) HPAI
A/chicken/England/030720/2020 (H5N8) HPAI
A/barnacle goose/Germany-SH/AI02167/2020 (H5N8) HPAI
A/mute swan/Netherlands/20015931-001/2020 (H5N8) HPAI
A/chicken/Luxembourg/21168413/2021 (H5N8) HPAI
A/Meleagris gallopavo/Belgium/11293 001/2021 (H5N8) HPAI
A/Chicken/Egypt/AI20286/2019 (H5N8) HPAI
A/chicken/Tatarstan/7/2018 (H5N8) HPAI
A/Great Black-backed Gull/Netherlands/1/2017 (H5N6) HPAI
A/Chicken/Egypt/AR553/2018 (H5N8) HPAI
A/tufted duck/Shimane/3211TY001/2017 (H5N6) HPAI
A/wild duck/South Korea/1801/2018 (H5N6) LPAI
A/Mallard(Anas platyrhynchos)/South Korea/KNU2019-23/2019 (H1N1)
A/Mallard(Anas platyrhynchos)/South Korea/KNU2019-50/2019 (H3N8)
A/Shoveler/Omsk Region/15/2019 (H4N6)
A/Mallard/Russia Primorje/153/2019 (H4N6)
A/Wild Duck/South Korea/KNU2020-101/2020 (H3N8)
A/goose/Tyumen/33-53/2021 (H5N1) HPAI
A/swan/France/21P012384/2021 (H5N1) HPAI
A/goose/Tyumen/33-52/2021 (H5N1) HPAI
A/chicken/Nigeria/VRD21-98 21VIR2288-6/2021 (H5N8) HPAI
A/mute swan/Slovakia/Pah6 21VIR1086-2/2021 (H5N5) HPAI
A/barnacle goose/Denmark/17572-1 01/2021 (H5N1) HPAI
A/barnacle goose/Netherlands/21027016-002/2021 (H5N1) HPAI
A/chicken/Kagoshima/21A6T/2021 (H5N1)
A/teal/Egypt/MB-D-487OP/2016 (H7N3) LPAI
A/duck/Bangladesh/41439/2019 (H7N3) LPAI
A/mallard/Korea/A44-5/2017 (H5N3) LPAI
A/wild bird/South Korea/WKU2019-4/2019 (H1N1)
A/duck/Guangdong/H31/2020 (H3N8)
A/wild duck/Korea/H214/2021 (H7N3) LPAI
A/duck/Nigeria/SK28T 19VIR8424-2/2019 (H5N6) HPAI
A/Branta leucopsis/Belgium/14735 0001/2021 (H5N1) HPAI
A/chicken/Netherlands/21037233-001/2021 (H5N1) HPAI
A/Mute swan/Netherlands/21037283-002/2021 (H5N1) HPAI
A/duck/Saratov/29-11/2021 (H5N1) HPAI
A/yellow-legged gull/Switzerland/15-0039 21VIR3035/2021 (H5N4) HPAI
A/buzzard/Germany-MV/AI02166/2020 (H5N5) HPAI
A/knot wader/Ireland/472 21VIR2956-1/2021 (H5N3) HPAI
A/mallard/Ukraine/AN-221-13-01/2020 (H7N2) LPAI
A/duck/Mongolia/961/2019 (H3N8)
A/goose/Czech Republic/20689-28T/2021 (H5N1) HPAI
A/Common Teal/Dagestan/34d/2019 (H4N6)
A/duck/Moscow/5743/2019 (H1N1)
A/Mallard/Netherlands/11/2021 (H5N2) LPAI
A/mallard/Italy/21VIR6957-6/2021 (H5N1) LPAI
A/guinea fowl/Germany-NW/AI01184/2020 (H5N8) LPAI
A/duck/Eastern China/008/2008 (H5N5) HPAI
A/Swan/Netherlands/14000281/2014 (H5N2) LPAI
A/duck/Mongolia/380/2019 (H4N6)
A/White-fronted goose(Anser albifrons)/South Korea/KNU2019-37/2019 (H1N1)
A/duck/Bangladesh/36391/2018 (H4N6)
A/duck/Bangladesh/37630/2019 (H10N4)
A/crane/Kagoshima/KU1/2014 (H5N8) HPAI
A/duck/Taiwan/A3400/2015 (H5N8) HPAI
A/Northern pintail/Washington/40964/2014 (H5N2) HPAI
A/crane/Kagoshima/KU41/2014 (H5N8) HPAI
A/tundra swan/Korea/H411/2014 (H5N8) HPAI
A/goose/Guangdong/k0103/2010 (H5N5) HPAI
A/duck/Eastern China/S1109/2014 (H5N8) HPAI
A/mallard/Shanghai/SH-9/2013 (H5N8) HPAI
A/duck/Vietnam/1151/2014 (H5N6) HPAI
A/pigeon/Sichuan/NCXN29/2014 (H5N1) HPAI
A/duck/Guangdong/GD01/2014 (H5N6) HPAI
A/Common pheasant/Hunan/11/2015 (H5N6) HPAI
A/Whooper swan/Mongolia/25/2020 (H5N6) HPAI
A/black swan/Akita/1/2016 (H5N6) HPAI
A/duck/Hyogo/1/2016 (H5N6) HPAI
A/chicken/England/11416/2008 (H7N7) HPAI
A/duck/Mongolia/520/2015 (H1N1)
A/Duck/Dongting/D76-1/2016 (H5N7) LPAI
A/Anas poecilorhyncha/South Korea/JB32-81/2019 (H11N2)
A/Common Teal/Buryatia/88i/2019 (H4N6)
A/duck/India/10CA01/2016 (H5N8) HPAI
A/great crested grebe/Uvs-Nuur Lake/341/2016 (H5N8) HPAI
A/grey-headed gull/Uganda/MUWRP-538/2017 (H5N8) HPAI
A/mallard/South Korea/34X-2/2021 (H7N7) LPAI
A/environment/Bangladesh/42635/2020 (H10N7)
A/mallard/South Korea/JB21-22/2019 (H4N6)
A/chicken/Germany/AR1386-L01302/2015 (H7N7) HPAI
A/mallard/Denmark/12947-12/2020-10-26 (H7N5) LPAI
A/Bar-headed Goose/Qinghai/a218/2017 (H5N8) HPAI
A/chicken/South Africa/499723/2018 (H5N8) HPAI
A/guinea fowl/Nigeria/OG-GF11T 19VIR8424-7/2019 (H5N8) HPAI
A/turkey/Czech Republic/3071/2020 (H5N8) HPAI
A/white-fronted goose/Germany-BB/AI00018/2020 (H5N8) HPAI
A/hawk/Poland/003/2020 (H5N8) HPAI
A/layer hen/Slovakia/A-chicken-Slovakia-Pah 14-2020 H5N8 PA/2020 (H5N8) HPAI
A/whooper swan/Fukushima/0701B002/2021 (H5N8) HPAI
A/white-tailed eagle/Hokkaido/20210127001/2021 (H5N8) HPAI
A/chicken/Kagawa/11C/2020 (H5N8) HPAI
A/environment/Kagoshima/KU-ngr-J2/2020 (H5N8) HPAI
A/Mandarin duck/Korea/H242/2020 (H5N8) HPAI
A/northern pintail/Hokkaido/M13/2020 (H5N8) HPAI
A/Mallard (Anas platyrhynchos)/South Korea/KNU2019-47/2019 (H4N6)
A/Mallard/Russia Primorje/129/2019 (H6N6)
A/Northern Pintail/Alaska/19TL00162/2019 (H3N8)
A/environment/Maryland/1162/2006 (H5N1) LPAI
A/Mallard/Ohio/19OS0177/2019 (H1N1)
A/ruddy turnstone/New Jersey/UGAI17-2072/2017 (H5N2) LPAI
A/environment/South Carolina/UGAI17-2243/2017 (H5N2) LPAI
A/Mallard/Ohio/18OS4660/2018 (H4N6)
A/Mallard duck/Alberta/147/2019 (H3N8)
0.02

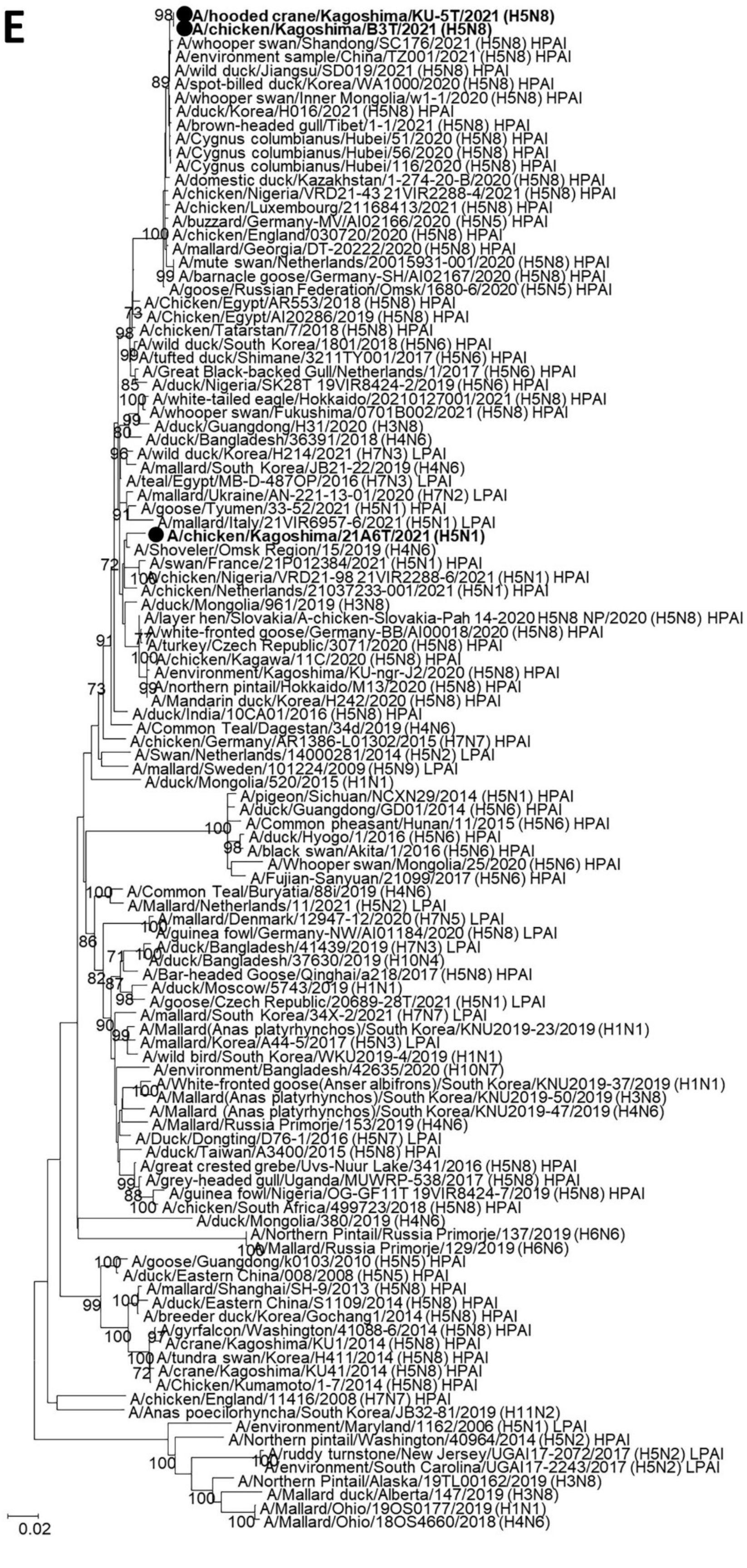
E
A/hooded crane/Kagoshima/KU-5T/2021 (H5N8)
A/chicken/Kagoshima/B3T/2021 (H5N8)
A/whooper swan/Shandong/SC176/2021 (H5N8) HPAI
A/environment sample/China/TZ001/2021 (H5N8) HPAI
A/wild duck/Jiangsu/SD019/2021 (H5N8) HPAI
A/spot-billed duck/Korea/WA1000/2020 (H5N8) HPAI
A/whooper swan/Inner Mongolia/w1-1/2020 (H5N8) HPAI
A/duck/Korea/H016/2021 (H5N8) HPAI
A/brown-headed gull/Tibet/1-1/2021 (H5N8) HPAI
A/Cygnus columbianus/Hubei/51/2020 (H5N8) HPAI
A/Cygnus columbianus/Hubei/56/2020 (H5N8) HPAI
A/Cygnus columbianus/Hubei/116/2020 (H5N8) HPAI
A/domestic duck/Kazakhstan/1-274-20-B/2020 (H5N8) HPAI
A/chicken/Nigeria/VRD21-43 21VIR2288-4/2021 (H5N8) HPAI
A/chicken/Luxembourg/21168413/2021 (H5N8) HPAI
A/buzzard/Germany-MV/AI02166/2020 (H5N5) HPAI
A/chicken/England/030720/2020 (H5N8) HPAI
A/mallard/Georgia/DT-20222/2020 (H5N8) HPAI
A/mute swan/Netherlands/20015931-001/2020 (H5N8) HPAI
A/barnacle goose/Germany-SH/AI02167/2020 (H5N8) HPAI
A/goose/Russian Federation/Omsk/1680-6/2020 (H5N5) HPAI
A/Chicken/Egypt/AR553/2018 (H5N8) HPAI
A/Chicken/Egypt/AI20286/2019 (H5N8) HPAI
A/chicken/Tatarstan/7/2018 (H5N8) HPAI
A/wild duck/South Korea/1801/2018 (H5N6) HPAI
A/tufted duck/Shimane/3211TY001/2017 (H5N6) HPAI
A/Great Black-backed Gull/Netherlands/1/2017 (H5N6) HPAI
A/duck/Nigeria/SK28T 19VIR8424-2/2019 (H5N6) HPAI
A/white-tailed eagle/Hokkaido/20210127001/2021 (H5N8) HPAI
A/whooper swan/Fukushima/0701B002/2021 (H5N8) HPAI
A/duck/Guangdong/H31/2020 (H3N8)
A/duck/Bangladesh/36391/2018 (H4N6)
A/wild duck/Korea/H214/2021 (H7N3) LPAI
A/mallard/South Korea/JB21-22/2019 (H4N6)
A/teal/Egypt/MB-D-487OP/2016 (H7N3) LPAI
A/mallard/Ukraine/AN-221-13-01/2020 (H7N2) LPAI
A/goose/Tyumen/33-52/2021 (H5N1) HPAI
A/mallard/Italy/21VIR6957-6/2021 (H5N1) LPAI
A/chicken/Kagoshima/21A6T/2021 (H5N1)
A/Shoveler/Omsk Region/15/2019 (H4N6)
A/swan/France/21P012384/2021 (H5N1) HPAI
A/chicken/Nigeria/VRD21-98 21VIR2288-6/2021 (H5N1) HPAI
A/chicken/Netherlands/21037233-001/2021 (H5N1) HPAI
A/duck/Mongolia/961/2019 (H3N8)
A/layer hen/Slovakia/A-chicken-Slovakia-Pah 14-2020 H5N8 NP/2020 (H5N8) HPAI
A/white-fronted goose/Germany-BB/AI00018/2020 (H5N8) HPAI
A/turkey/Czech Republic/3071/2020 (H5N8) HPAI
A/chicken/Kagawa/11C/2020 (H5N8) HPAI
A/environment/Kagoshima/KU-ngr-J2/2020 (H5N8) HPAI
A/northern pintail/Hokkaido/M13/2020 (H5N8) HPAI
A/Mandarin duck/Korea/H242/2020 (H5N8) HPAI
A/duck/India/10CA01/2016 (H5N8) HPAI
A/Common Teal/Dagestan/34d/2019 (H4N6)
A/chicken/Germany/AR1386-L01302/2015 (H7N7) HPAI
A/Swan/Netherlands/14000281/2014 (H5N2) LPAI
A/mallard/Sweden/101224/2009 (H5N9) LPAI
A/duck/Mongolia/520/2015 (H1N1)
A/pigeon/Sichuan/NCXN29/2014 (H5N1) HPAI
A/duck/Guangdong/GD01/2014 (H5N6) HPAI
A/Common pheasant/Hunan/11/2015 (H5N6) HPAI
A/duck/Hyogo/1/2016 (H5N6) HPAI
A/black swan/Akita/1/2016 (H5N6) HPAI
A/Whooper swan/Mongolia/25/2020 (H5N6) HPAI
A/Fujian-Sanyuan/21099/2017 (H5N6) HPAI
A/Common Teal/Buryatia/88i/2019 (H4N6)
A/Mallard/Netherlands/11/2021 (H5N2) LPAI
A/mallard/Denmark/12947-12/2020 (H7N5) LPAI
A/guinea fowl/Germany-NW/AI01184/2020 (H5N8) LPAI
A/duck/Bangladesh/41439/2019 (H7N3) LPAI
A/duck/Bangladesh/37630/2019 (H10N4)
A/Bar-headed Goose/Qinghai/a218/2017 (H5N8) HPAI
A/duck/Moscow/5743/2019 (H1N1)
A/goose/Czech Republic/20689-28T/2021 (H5N1) LPAI
A/mallard/South Korea/34X-2/2021 (H7N7) LPAI
A/Mallard(Anas platyrhynchos)/South Korea/KNU2019-23/2019 (H1N1)
A/mallard/Korea/A44-5/2017 (H5N3) LPAI
A/wild bird/South Korea/WKU2019-4/2019 (H1N1)
A/environment/Bangladesh/42635/2020 (H10N7)
A/White-fronted goose(Anser albifrons)/South Korea/KNU2019-37/2019 (H1N1)
A/Mallard(Anas platyrhynchos)/South Korea/KNU2019-50/2019 (H3N8)
A/Mallard (Anas platyrhynchos)/South Korea/KNU2019-47/2019 (H4N6)
A/Mallard/Russia Primorje/153/2019 (H4N6)
A/Duck/Dongting/D76-1/2016 (H5N7) LPAI
A/duck/Taiwan/A3400/2015 (H5N8) HPAI
A/great crested grebe/Uvs-Nuur Lake/341/2016 (H5N8) HPAI
A/grey-headed gull/Uganda/MUWRP-538/2017 (H5N8) HPAI
A/guinea fowl/Nigeria/OG-GF11T 19VIR8424-7/2019 (H5N8) HPAI
A/chicken/South Africa/499723/2018 (H5N8) HPAI
A/duck/Mongolia/380/2019 (H4N6)
A/Northern Pintail/Russia Primorje/137/2019 (H6N6)
A/Mallard/Russia Primorje/129/2019 (H6N6)
A/goose/Guangdong/k0103/2010 (H5N5) HPAI
A/duck/Eastern China/008/2008 (H5N5) HPAI
A/mallard/Shanghai/SH-9/2013 (H5N8) HPAI
A/duck/Eastern China/S1109/2014 (H5N8) HPAI
A/breeder duck/Korea/Gochang1/2014 (H5N8) HPAI
A/gyrfalcon/Washington/41088-6/2014 (H5N8) HPAI
A/crane/Kagoshima/KU1/2014 (H5N8) HPAI
A/tundra swan/Korea/H411/2014 (H5N8) HPAI
A/crane/Kagoshima/KU41/2014 (H5N8) HPAI
A/Chicken/Kumamoto/1-7/2014 (H5N8) HPAI
A/chicken/England/11416/2008 (H7N7) HPAI
A/Anas poecilorhyncha/South Korea/JB32-81/2019 (H11N2)
A/environment/Maryland/1162/2006 (H5N1) LPAI
A/Northern pintail/Washington/40964/2014 (H5N2) HPAI
A/ruddy turnstone/New Jersey/UGAI17-2072/2017 (H5N2) LPAI
A/environment/South Carolina/UGAI17-2243/2017 (H5N2) LPAI
A/Northern Pintail/Alaska/19TL00162/2019 (H3N8)
A/Mallard duck/Alberta/147/2019 (H3N8)
A/Mallard/Ohio/19OS0177/2019 (H1N1)
A/Mallard/Ohio/18OS4660/2018 (H4N6)
0.02

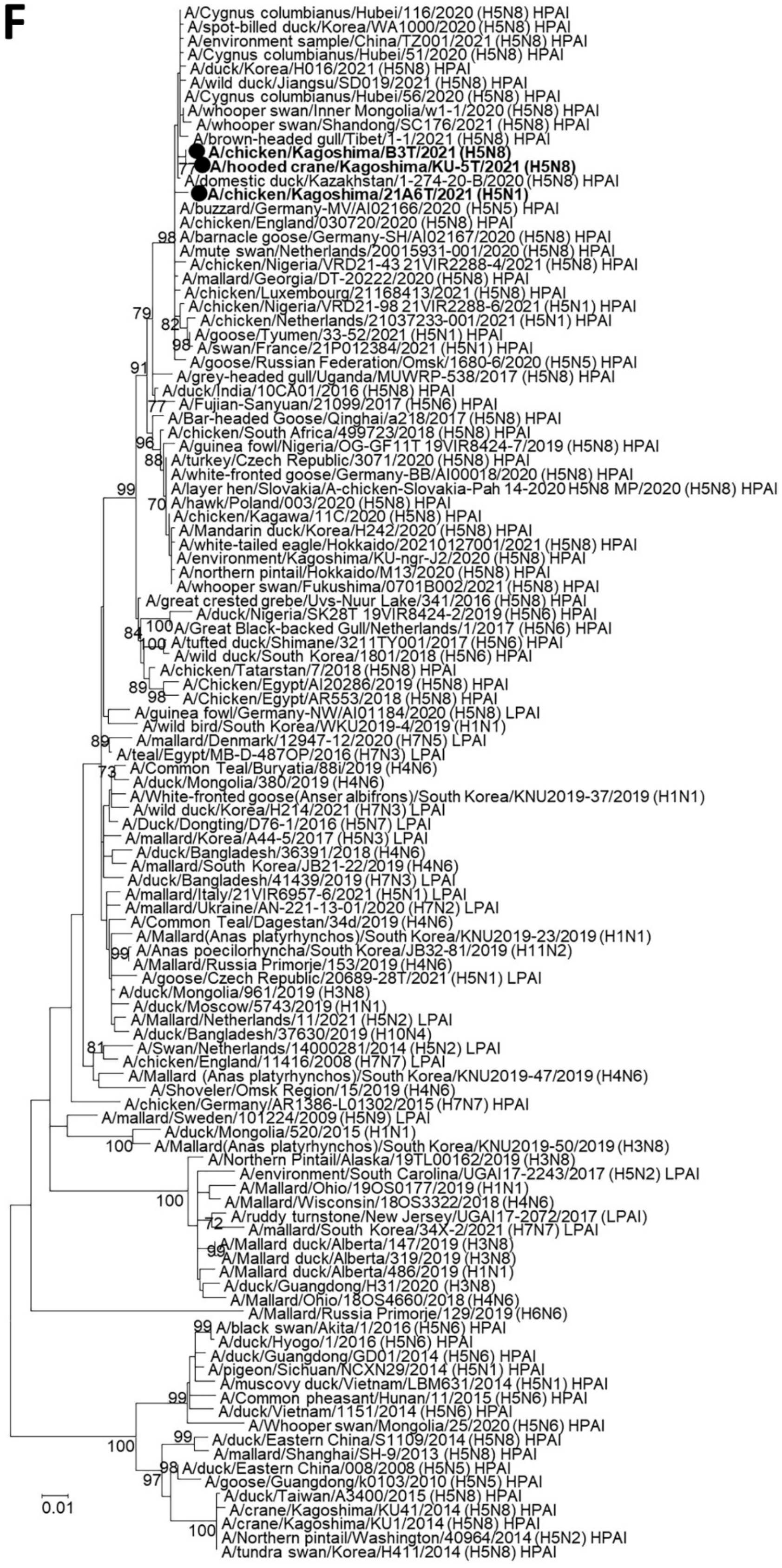
F
A/Cygnus columbianus/Hubei/116/2020 (H5N8) HPAI
A/spot-billed duck/Korea/WA1000/2020 (H5N8) HPAI
A/environment sample/China/TZ001/2021 (H5N8) HPAI
A/Cygnus columbianus/Hubei/51/2020 (H5N8) HPAI
A/duck/Korea/H016/2021 (H5N8) HPAI
A/wild duck/Jiangsu/SD019/2021 (H5N8) HPAI
A/Cygnus columbianus/Hubei/56/2020 (H5N8) HPAI
A/whooper swan/Inner Mongolia/w1-1/2020 (H5N8) HPAI
A/whooper swan/Shandong/SC176/2021 (H5N8) HPAI
A/brown-headed gull/Tibet/1-1/2021 (H5N8) HPAI
A/chicken/Kagoshima/B3T/2021 (H5N8)
A/hooded crane/Kagoshima/KU-5T/2021 (H5N8)
A/domestic duck/Kazakhstan/1-274-20-B/2020 (H5N8) HPAI
A/chicken/Kagoshima/21A6T/2021 (H5N1)
A/buzzard/Germany-MV/AI02166/2020 (H5N5) HPAI
A/chicken/England/030720/2020 (H5N8) HPAI
A/barnacle goose/Germany-SH/AI02167/2020 (H5N8) HPAI
A/mute swan/Netherlands/20015931-001/2020 (H5N8) HPAI
A/chicken/Nigeria/VRD21-43 21VIR2288-4/2021 (H5N8) HPAI
A/mallard/Georgia/DT-20222/2020 (H5N8) HPAI
A/chicken/Luxembourg/21168413/2021 (H5N8) HPAI
A/chicken/Nigeria/VRD21-98 21VIR2288-6/2021 (H5N1) HPAI
A/chicken/Netherlands/21037233-001/2021 (H5N1) HPAI
A/goose/Tyumen/33-52/2021 (H5N1) HPAI
A/swan/France/21P012384/2021 (H5N1) HPAI
A/goose/Russian Federation/Omsk/1680-6/2020 (H5N5) HPAI
A/grey-headed gull/Uganda/MUWRP-538/2017 (H5N8) HPAI
A/duck/India/10CA01/2016 (H5N8) HPAI
A/Fujian-Sanyuan/21099/2017 (H5N6) HPAI
A/Bar-headed Goose/Qinghai/a218/2017 (H5N8) HPAI
A/chicken/South Africa/499723/2018 (H5N8) HPAI
A/guinea fowl/Nigeria/OG-GF11T 19VIR8424-7/2019 (H5N8) HPAI
A/turkey/Czech Republic/3071/2020 (H5N8) HPAI
A/white-fronted goose/Germany-BB/AI00018/2020 (H5N8) HPAI
A/layer hen/Slovakia/A-chicken-Slovakia-Pah 14-2020 H5N8 MP/2020 (H5N8) HPAI
A/hawk/Poland/003/2020 (H5N8) HPAI
A/chicken/Kagawa/11C/2020 (H5N8) HPAI
A/Mandarin duck/Korea/H242/2020 (H5N8) HPAI
A/white-tailed eagle/Hokkaido/20210127001/2021 (H5N8) HPAI
A/environment/Kagoshima/KU-ngr-J2/2020 (H5N8) HPAI
A/northern pintail/Hokkaido/M13/2020 (H5N8) HPAI
A/whooper swan/Fukushima/0701B002/2021 (H5N8) HPAI
A/great crested grebe/Uvs-Nuur Lake/341/2016 (H5N8) HPAI
A/duck/Nigeria/SK28T 19VIR8424-2/2019 (H5N6) HPAI
A/Great Black-backed Gull/Netherlands/1/2017 (H5N6) HPAI
A/tufted duck/Shimane/3211TY001/2017 (H5N6) HPAI
A/wild duck/South Korea/1801/2018 (H5N6) HPAI
A/chicken/Tatarstan/7/2018 (H5N8) HPAI
A/Chicken/Egypt/AI20286/2019 (H5N8) HPAI
A/Chicken/Egypt/AR553/2018 (H5N8) HPAI
A/guinea fowl/Germany-NW/AI01184/2020 (H5N8) LPAI
A/wild bird/South Korea/WKU2019-4/2019 (H1N1)
A/mallard/Denmark/12947-12/2020 (H7N5) LPAI
A/teal/Egypt/MB-D-487OP/2016 (H7N3) LPAI
A/Common Teal/Buryatia/88i/2019 (H4N6)
A/duck/Mongolia/380/2019 (H4N6)
A/White-fronted goose(Anser albifrons)/South Korea/KNU2019-37/2019 (H1N1)
A/wild duck/Korea/H214/2021 (H7N3) LPAI
A/Duck/Dongting/D76-1/2016 (H5N7) LPAI
A/mallard/Korea/A44-5/2017 (H5N3) LPAI
A/duck/Bangladesh/36391/2018 (H4N6)
A/mallard/South Korea/JB21-22/2019 (H4N6)
A/duck/Bangladesh/41439/2019 (H7N3) LPAI
A/mallard/Italy/21VIR6957-6/2021 (H5N1) LPAI
A/mallard/Ukraine/AN-221-13-01/2020 (H7N2) LPAI
A/Common Teal/Dagestan/34d/2019 (H4N6)
A/Mallard(Anas platyrhynchos)/South Korea/KNU2019-23/2019 (H1N1)
A/Anas poecilorhyncha/South Korea/JB32-81/2019 (H11N2)
A/Mallard/Russia Primorje/153/2019 (H4N6)
A/goose/Czech Republic/20689-28T/2021 (H5N1) LPAI
A/duck/Mongolia/961/2019 (H3N8)
A/duck/Moscow/5743/2019 (H1N1)
A/Mallard/Netherlands/11/2021 (H5N2) LPAI
A/duck/Bangladesh/37630/2019 (H10N4)
A/Swan/Netherlands/14000281/2014 (H5N2) LPAI
A/chicken/England/11416/2008 (H7N7) LPAI
A/Mallard (Anas platyrhynchos)/South Korea/KNU2019-47/2019 (H4N6)
A/Shoveler/Omsk Region/15/2019 (H4N6)
A/chicken/Germany/AR1386-L01302/2015 (H7N7) HPAI
A/mallard/Sweden/101224/2009 (H5N9) LPAI
A/duck/Mongolia/520/2015 (H1N1)
A/Mallard(Anas platyrhynchos)/South Korea/KNU2019-50/2019 (H3N8)
A/Northern Pintail/Alaska/19TL00162/2019 (H3N8)
A/environment/South Carolina/UGAI17-2243/2017 (H5N2) LPAI
A/Mallard/Ohio/19OS0177/2019 (H1N1)
A/Mallard/Wisconsin/18OS3322/2018 (H4N6)
A/ruddy turnstone/New Jersey/UGAI17-2072/2017 (LPAI)
A/mallard/South Korea/34X-2/2021 (H7N7) LPAI
A/Mallard duck/Alberta/147/2019 (H3N8)
A/Mallard duck/Alberta/319/2019 (H3N8)
A/Mallard duck/Alberta/486/2019 (H1N1)
A/duck/Guangdong/H31/2020 (H3N8)
A/Mallard/Ohio/18OS4660/2018 (H4N6)
A/Mallard/Russia Primorje/129/2019 (H6N6)
A/black swan/Akita/1/2016 (H5N6) HPAI
A/duck/Hyogo/1/2016 (H5N6) HPAI
A/duck/Guangdong/GD01/2014 (H5N6) HPAI
A/pigeon/Sichuan/NCXN29/2014 (H5N1) HPAI
A/muscovy duck/Vietnam/LBM631/2014 (H5N1) HPAI
A/Common pheasant/Hunan/11/2015 (H5N6) HPAI
A/duck/Vietnam/1151/2014 (H5N6) HPAI
A/Whooper swan/Mongolia/25/2020 (H5N6) HPAI
A/duck/Eastern China/S1109/2014 (H5N8) HPAI
A/mallard/Shanghai/SH-9/2013 (H5N8) HPAI
A/duck/Eastern China/008/2008 (H5N5) HPAI
A/goose/Guangdong/k0103/2010 (H5N5) HPAI
A/duck/Taiwan/A3400/2015 (H5N8) HPAI
A/crane/Kagoshima/KU41/2014 (H5N8) HPAI
A/crane/Kagoshima/KU1/2014 (H5N8) HPAI
A/Northern pintail/Washington/40964/2014 (H5N2) HPAI
A/tundra swan/Korea/H411/2014 (H5N8) HPAI
0.01

G

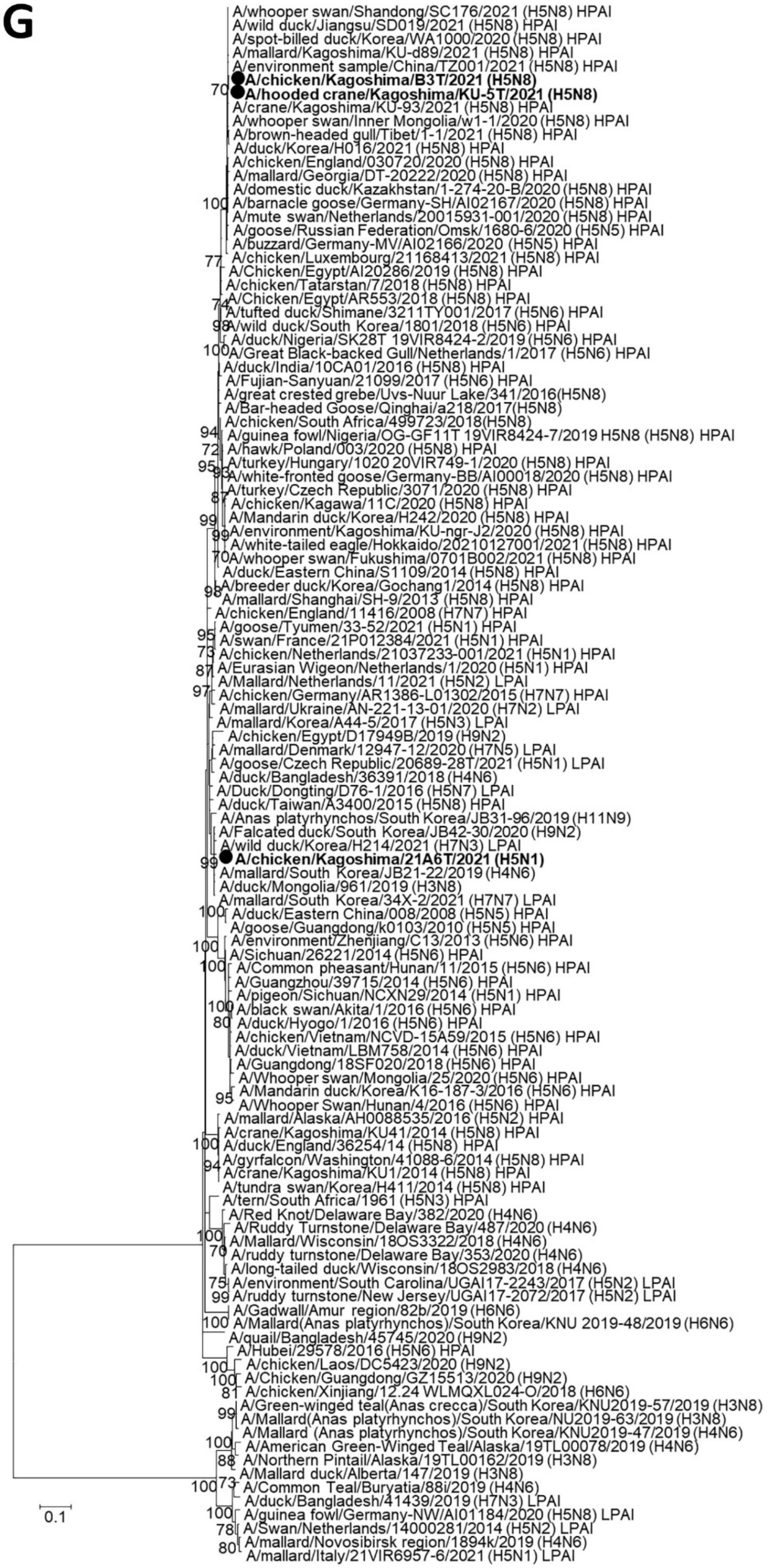
A/whooper swan/Shandong/SC176/2021 (H5N8) HPAI
A/wild duck/Jiangsu/SD019/2021 (H5N8) HPAI
A/spot-billed duck/Korea/WA1000/2020 (H5N8) HPAI
A/mallard/Kagoshima/KU-d89/2021 (H5N8) HPAI
A/environment sample/China/TZ001/2021 (H5N8) HPAI
A/chicken/Kagoshima/B3T/2021 (H5N8)
A/hooded crane/Kagoshima/KU-5T/2021 (H5N8)
A/crane/Kagoshima/KU-93/2021 (H5N8) HPAI
A/whooper swan/Inner Mongolia/w1-1/2020 (H5N8) HPAI
A/brown-headed gull/Tibet/1-1/2021 (H5N8) HPAI
A/duck/Korea/H016/2021 (H5N8) HPAI
A/chicken/England/030720/2020 (H5N8) HPAI
A/mallard/Georgia/DT-20222/2020 (H5N8) HPAI
A/domestic duck/Kazakhstan/1-274-20-B/2020 (H5N8) HPAI
A/barnacle goose/Germany-SH/AI02167/2020 (H5N8) HPAI
A/mute swan/Netherlands/20015931-001/2020 (H5N8) HPAI
A/goose/Russian Federation/Omsk/1680-6/2020 (H5N5) HPAI
A/buzzard/Germany-MV/AI02166/2020 (H5N5) HPAI
A/chicken/Luxembourg/21168413/2021 (H5N8) HPAI
A/Chicken/Egypt/AI20286/2019 (H5N8) HPAI
A/chicken/Tatarstan/7/2018 (H5N8) HPAI
A/Chicken/Egypt/AR553/2018 (H5N8) HPAI
A/tufted duck/Shimane/3211TY001/2017 (H5N6) HPAI
A/wild duck/South Korea/1801/2018 (H5N6) HPAI
A/duck/Nigeria/SK28T 19VIR8424-2/2019 (H5N6) HPAI
A/Great Black-backed Gull/Netherlands/1/2017 (H5N6) HPAI
A/duck/India/10CA01/2016 (H5N8) HPAI
A/Fujian-Sanyuan/21099/2017 (H5N6) HPAI
A/great crested grebe/Uvs-Nuur Lake/341/2016(H5N8)
A/Bar-headed Goose/Qinghai/a218/2017(H5N8)
A/chicken/South Africa/499723/2018(H5N8)
A/guinea fowl/Nigeria/OG-GF11T 19VIR8424-7/2019 H5N8 (H5N8) HPAI
A/hawk/Poland/003/2020 (H5N8) HPAI
A/turkey/Hungary/1020 20VIR749-1/2020 (H5N8) HPAI
A/white-fronted goose/Germany-BB/AI00018/2020 (H5N8) HPAI
A/turkey/Czech Republic/3071/2020 (H5N8) HPAI
A/chicken/Kagawa/11C/2020 (H5N8) HPAI
A/Mandarin duck/Korea/H242/2020 (H5N8) HPAI
A/environment/Kagoshima/KU-ngr-J2/2020 (H5N8) HPAI
A/white-tailed eagle/Hokkaido/20210127001/2021 (H5N8) HPAI
A/whooper swan/Fukushima/0701B002/2021 (H5N8) HPAI
A/duck/Eastern China/S1109/2014 (H5N8) HPAI
A/breeder duck/Korea/Gochang1/2014 (H5N8) HPAI
A/mallard/Shanghai/SH-9/2013 (H5N8) HPAI
A/chicken/England/11416/2008 (H7N7) HPAI
A/goose/Tyumen/33-52/2021 (H5N1) HPAI
A/swan/France/21P012384/2021 (H5N1) HPAI
A/chicken/Netherlands/21037233-001/2021 (H5N1) HPAI
A/Eurasian Wigeon/Netherlands/1/2020 (H5N1) HPAI
A/Mallard/Netherlands/11/2021 (H5N2) LPAI
A/chicken/Germany/AR1386-L01302/2015 (H7N7) HPAI
A/mallard/Ukraine/AN-221-13-01/2020 (H7N2) LPAI
A/mallard/Korea/A44-5/2017 (H5N3) LPAI
A/chicken/Egypt/D17949B/2019 (H9N2)
A/mallard/Denmark/12947-12/2020 (H7N5) LPAI
A/goose/Czech Republic/20689-28T/2021 (H5N1) LPAI
A/duck/Bangladesh/36391/2018 (H4N6)
A/Duck/Dongting/D76-1/2016 (H5N7) LPAI
A/duck/Taiwan/A3400/2015 (H5N8) HPAI
A/Anas platyrhynchos/South Korea/JB31-96/2019 (H11N9)
A/Falcated duck/South Korea/JB42-30/2020 (H9N2)
A/wild duck/Korea/H214/2021 (H7N3) LPAI
A/chicken/Kagoshima/21A6T/2021 (H5N1)
A/mallard/South Korea/JB21-22/2019 (H4N6)
A/duck/Mongolia/961/2019 (H3N8)
A/mallard/South Korea/34X-2/2021 (H7N7) LPAI
A/duck/Eastern China/008/2008 (H5N5) HPAI
A/goose/Guangdong/k0103/2010 (H5N5) HPAI
A/environment/Zhenjiang/C13/2013 (H5N6) HPAI
A/Sichuan/26221/2014 (H5N6) HPAI
A/Common pheasant/Hunan/11/2015 (H5N6) HPAI
A/Guangzhou/39715/2014 (H5N6) HPAI
A/pigeon/Sichuan/NCXN29/2014 (H5N1) HPAI
A/black swan/Akita/1/2016 (H5N6) HPAI
A/duck/Hyogo/1/2016 (H5N6) HPAI
A/chicken/Vietnam/NCVD-15A59/2015 (H5N6) HPAI
A/duck/Vietnam/LBM758/2014 (H5N6) HPAI
A/Guangdong/18SF020/2018 (H5N6) HPAI
A/Whooper swan/Mongolia/25/2020 (H5N6) HPAI
A/Mandarin duck/Korea/K16-187-3/2016 (H5N6) HPAI
A/Whooper Swan/Hunan/4/2016 (H5N6) HPAI
A/mallard/Alaska/AH0088535/2016 (H5N2) HPAI
A/crane/Kagoshima/KU41/2014 (H5N8) HPAI
A/duck/England/36254/14 (H5N8) HPAI
A/gyrfalcon/Washington/41088-6/2014 (H5N8) HPAI
A/crane/Kagoshima/KU1/2014 (H5N8) HPAI
A/tundra swan/Korea/H411/2014 (H5N8) HPAI
A/tern/South Africa/1961 (H5N3) HPAI
A/Red Knot/Delaware Bay/382/2020 (H4N6)
A/Ruddy Turnstone/Delaware Bay/487/2020 (H4N6)
A/Mallard/Wisconsin/18OS3322/2018 (H4N6)
A/ruddy turnstone/Delaware Bay/353/2020 (H4N6)
A/long-tailed duck/Wisconsin/18OS2983/2018 (H4N6)
A/environment/South Carolina/UGAI17-2243/2017 (H5N2) LPAI
A/ruddy turnstone/New Jersey/UGAI17-2072/2017 (H5N2) LPAI
A/Gadwall/Amur region/82b/2019 (H6N6)
A/Mallard(Anas platyrhynchos)/South Korea/KNU 2019-48/2019 (H6N6)
A/quail/Bangladesh/45745/2020 (H9N2)
A/Hubei/29578/2016 (H5N6) HPAI
A/chicken/Laos/DC5423/2020 (H9N2)
A/Chicken/Guangdong/GZ15513/2020 (H9N2)
A/chicken/Xinjiang/12.24 WLMQXL024-O/2018 (H6N6)
A/Green-winged teal(Anas crecca)/South Korea/KNU2019-57/2019 (H3N8)
A/Mallard(Anas platyrhynchos)/South Korea/NU2019-63/2019 (H3N8)
A/Mallard (Anas platyrhynchos)/South Korea/KNU2019-47/2019 (H4N6)
A/American Green-Winged Teal/Alaska/19TL00078/2019 (H4N6)
A/Northern Pintail/Alaska/19TL00162/2019 (H3N8)
A/Mallard duck/Alberta/147/2019 (H3N8)
A/Common Teal/Buryatia/88i/2019 (H4N6)
A/duck/Bangladesh/41439/2019 (H7N3) LPAI
A/guinea fowl/Germany-NW/AI01184/2020 (H5N8) LPAI
A/Swan/Netherlands/14000281/2014 (H5N2) LPAI
A/mallard/Novosibirsk region/1894k/2019 (H4N6)
A/mallard/Italy/21VIR6957-6/2021 (H5N1) LPAI
0.1

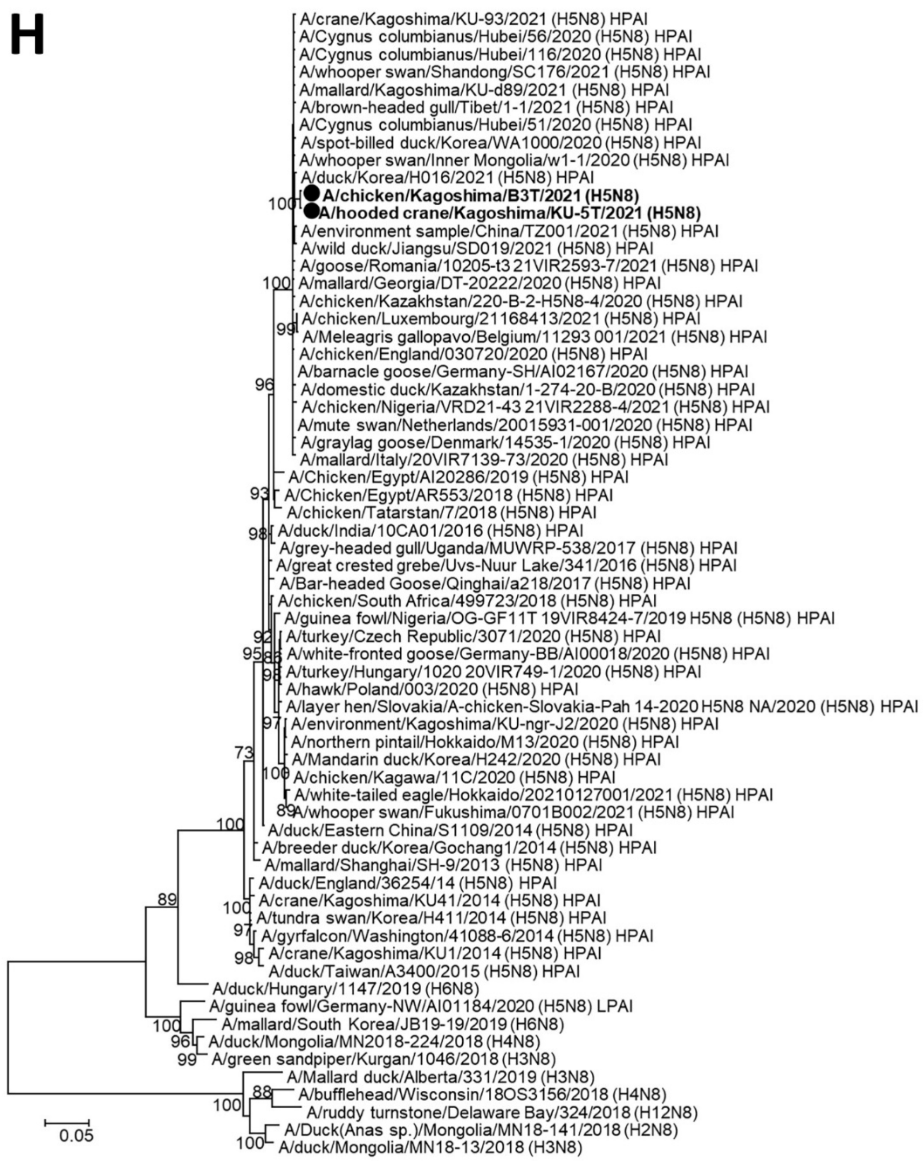

Appendix Figure 1. Phylogenetic tree of hemagglutinin genes of highly pathogenic avian influenza A(H5N1/H5N8) viruses isolated among wild waterfowl and domestic poultry on the Izumi plain, Japan in November 2021. We phylogenetically analyzed the nucleotide sequences of the genes from A/environment/Kagoshima/KU-ngrB4/2021 (mixed), A/chicken/Kagoshima/21A6T/2021 (H5N1), A/chicken/Kagoshima/B3T/2021 (H5N8), and A/hooded crane/Kagoshima/KU-5T/2021 (H5N8) with representative counterparts by using the maximum-likelihood method with a bootstrapping set of 1,000 replicates. Black circles with bold text indicate viruses isolated in this study. Area inside dotted box indicates genetic group 2 shown in Figure 2. Bootstrap values >70% are shown at the nodes. Scale bar indicates nucleotide substitutions per site. HPAI, highly pathogenic avian influenza; LPAI, low pathogenicity avian influenza.

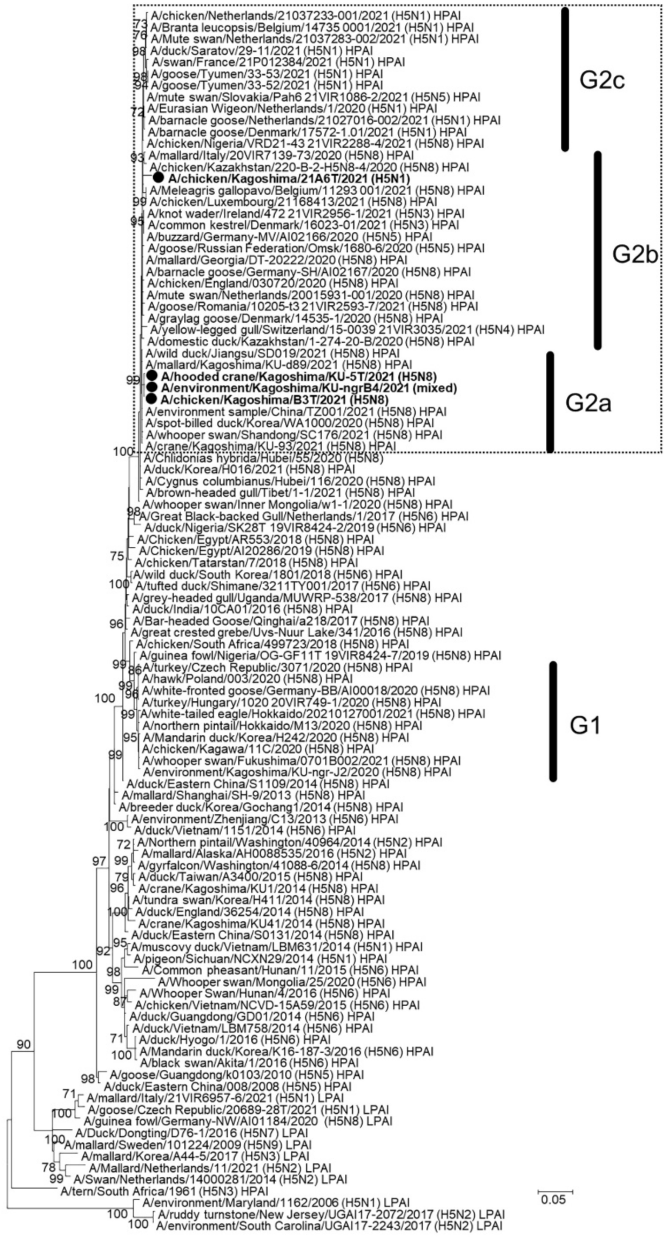

Appendix Figure 2. Phylogenetic trees of genes from highly pathogenic avian influenza A(H5N1/H5N8) viruses isolated on the Izumi plain, Japan in November 2021. Trees represent H5N1 genes NA (A), PB2 (B), PB1 (C), PA (D), NP (E), M (F), and NSP (G); and the H5N8 NA gene (H). Genes from A/chicken/Kagoshima/21A6T/2021 (H5N1), A/chicken/Kagoshima/B3T/2021 (H5N8), and A/hooded crane/Kagoshima/KU-5T/2021 (H5N8) were phylogenetically analyzed with representative counterparts using the maximum-likelihood method with a bootstrapping set of 1,000 replicates. Black circles and bold text indicate viruses isolated in this study. Bootstrap values >70% are shown at the nodes. Scale bar indicates nucleotide substitutions per site. HA, hemagglutinin; HPAI, highly pathogenic avian influenza; LPAI, low pathogenicity avian influenza; M, matrix; NA, neuraminidase; NP, nucleoprotein; NSP, nonstructural protein; PA, polymerase; PB1, polymerase basic 1; PB2, polymerase basic 2.
